# Screening for iron deficiency in young women: the predictive validity of a five-item screening instrument (IRON-5)

**DOI:** 10.1080/02813432.2026.2649329

**Published:** 2026-03-31

**Authors:** Fredrik Vinge, Anna Stubbendorff, Beata Borgström Bolmsjö, Ulf Jakobsson, Veronica Milos Nymberg, Moa Wolff

**Affiliations:** a Centre for Primary Health Care Research, Department of Clinical Sciences Malmö, Lund University, Malmö, Sweden; b University Clinic Primary Care, Skåne University Hospital, Region Skåne, Sweden; c Nutritional Epidemiology, Department of Clinical Sciences Malmö, Lund University, Malmö, Sweden

**Keywords:** Iron deficiency, adolescent, female, primary health care, risk assessment, mass screening, dietary habits

## Abstract

**Background:**

Iron deficiency is the most common micronutrient deficiency worldwide, negatively affecting quality of life and health. Young women are at particularly high risk due to increased iron demands during growth, menstrual blood losses, and dietary habits. There are no official guidelines recommending screening for iron deficiency in healthy young women. This study aimed to develop a screening instrument (IRON-5) to predict iron deficiency in this group, with relevance for use in primary care and school health services.

**Methods:**

In 2023, a cross-sectional study was conducted to assess the prevalence of iron deficiency and associated factors in high school girls. A questionnaire assessing dietary habits, menstrual bleeding patterns, iron supplement use, and hormonal contraceptive use was administered. Iron deficiency was defined as serum ferritin <15 µg/L. A post-hoc analysis identified the strongest predictors of iron deficiency and incorporated them into a risk-scoring model.

**Results:**

The final instrument included five predictors: a non-omnivorous diet, heavy menstruation causing discomfort, avoidance of activities due to menstruation, no iron supplementation, and non-use of hormonal contraceptives. A scoring system (0–5) was developed. Using a threshold score of ≥ 3, the model achieved a sensitivity of 74% and specificity of 57% for detecting iron deficiency.

**Conclusion:**

The IRON-5 is a concise and user-friendly screening instrument designed to identify young women at risk of iron deficiency. It demonstrated good sensitivity and may serve as a cost-effective method to prioritize individuals for blood testing. IRON-5 could support identification and prevention strategies in school-based and primary care settings.

## Introduction

Anaemia is a significant global health issue, with a prevalence of 24.3% across all age groups, resulting in 52 million Years Lived with Disability (YLD) in 2021. The leading cause of anaemia is iron deficiency [1]. Ferritin has been identified as the best blood test for iron deficiency and is recommended by the World Health Organization (WHO) [2,3]. Adolescent females are particularly at risk of iron deficiency due to increased iron demands during puberty, menstrual blood loss, and insufficient dietary intake [4]. Consequently, iron deficiency is significantly more prevalent among young women than men and may contribute to reduced perceived health in this population [5,6]. In Sweden, the prevalence of iron deficiency in adolescent females increased from 37% in 1994 to 45% in 2002, a rise that coincided with the discontinuation of iron fortification in flour [7]. A growing trend of reduced meat consumption, particularly among young people may further exacerbate this issue, as red meat is a major source of haem iron, a more bioavailable form [8,9]. While this trend raises nutritional concerns, it is not solely negative. Diets that include less red meat and more plant-based foods have been associated with significant health and environmental benefits. Such dietary patterns can reduce the risk of chronic diseases and substantially lower greenhouse gas emissions, thereby contributing to improved public health and enhanced environmental sustainability [10].

Emerging evidence suggests that even mild to moderate iron deficiency, without anaemia, can adversely affect physical performance, cognitive function, work capacity, and overall health [11–14]. In a systematic review the authors concluded that intravenous iron reduced fatigue in patients with iron deficiency without anaemia [15]. While treatment benefits have primarily been demonstrated in symptomatic individuals, it remains unclear whether iron supplementation provides similar benefits when offered to screen-detected individuals without overt symptoms. However, in light of the substantial deterioration in mental health among Swedish adolescent girls over the past two decades, including increasing levels of fatigue, stress-related symptoms, and self-reported poor health, the concept of being ‘asymptomatic’ becomes less clear-cut. Many of these commonly reported complaints overlap with symptoms potentially associated with iron deficiency, even in the absence of anaemia. At present, the role of dietary changes and undetected or untreated iron deficiency in contributing to this impaired well-being remains insufficiently understood [16].

There are many recommendations for iron deficiency screening programs on infants and patients with chronic kidney disease, inflammatory bowel disease, cardiac disease, and pregnant women [17–20]. While previous research supports screening for iron deficiency in healthy women of reproductive age, no official guidelines exist for this population [21]. A risk-based questionnaire approach to prioritize individuals for confirmatory testing is broadly consistent with the Wilson and Jungner principles of screening [22].

The aetiology of iron deficiency is multifaceted and will result from any condition in which the iron demands cannot be met by dietary iron intake. The most common cause of iron loss in young women is menstrual bleeding, which can be effectively reduced by the use of hormonal contraceptives [23–25]. Dietary habits are a critical factor for developing iron deficiency and long-term consumption of a vegan diet is associated with lower iron concentrations [26,27]. The Iron Insight Study, a cross-sectional study conducted in 2023, found that omnivores had higher ferritin levels compared to vegans/vegetarians, pescatarians and individuals who do not consume red meat [28]. The study also identified an association between heavy menstrual bleeding and iron deficiency [29].

This study sought to explore the potential of a questionnaire to predict iron deficiency, focusing on the questions that were most strongly associated with low ferritin levels in this cohort. The aim was to develop a concise and effective screening instrument to predict the risk of iron deficiency in young women.

## Methods

### Study design

This study is a post hoc analysis of the Iron Insight study, a cross-sectional study conducted in October 2023. The Iron Insight study assessed the prevalence of iron deficiency in a population of adolescent girls and explored potential associations with dietary habits, menstruation, contraceptive use, and use of iron-containing supplements [28]. The present analysis aimed to develop a screening instrument for iron deficiency by identifying the combination of questions that maximized sensitivity and specificity in detecting iron deficiency. Iron deficiency was defined solely by serum ferritin <15 µg/L, independent of haemoglobin levels. To reduce the risk of outcome misclassification, participants with markedly elevated serum ferritin (>150 µg/L) were excluded from the analytic sample. Participants with iron deficiency anaemia were included and analysed together with those with isolated iron deficiency, as the target condition of interest was iron deficiency irrespective of anaemia.

### Setting

Female high school students aged 16–19 years were recruited from two high schools in Malmö and Lund, Sweden, through advertisements in the school area and information provided by teachers. Students who were willing to participate were invited to the school assembly hall for a general information session, followed by data collection for those who consented to participate.

Inclusion criteria were age ≥15 years and post-menarcheal status. Exclusion criteria were chronic inflammatory disease, ongoing bacterial infection, and pregnancy. Ongoing bacterial infection was assessed by self-report; participants were asked whether they were currently receiving antibiotic treatment for an infection, and those reporting ongoing antibiotic treatment were excluded.

A digital questionnaire was used to collect data on dietary habits, menstrual bleeding patterns, and background characteristics. No inflammatory markers, such as C-reactive protein (CRP), were measured.

Dietary habits among the participants were assessed through an online dietary document. The participants classified their dietary habits (last year) as omnivore, excluding red meat, pescatarian, vegetarian, or vegan. For our analysis, these categories were merged into two groups: omnivores and non-omnivores (non-consumers of red meat, pescatarians, vegetarians, and vegans).

Menstrual bleeding patterns were assessed using the validated 6-item SAMANTA questionnaire. Since no Swedish translation was available, a Swedish version of the questionnaire was developed through back-translation in collaboration with a professional English editor. The SAMANTA questionnaire consists of the following questions:

1. Do you experience menstrual bleeding for more than seven days per month?
2. Do you experience three or more days of heavier menstrual bleeding during your menstrual period?
3. In general, does menstruation bother you due to its abundance?
4. During any of these heavier menstrual bleeding days, do you spot your clothes at night; or would you spot them if you did not use double protection/did not change your clothes during the night?
5. During these heavier menstrual days, are you worried about staining the chair, sofa, etc.?
6. In general, during these heavier menstrual bleeding days, do you avoid, as far as possible, some activities, trips, or leisure-time plans because you frequently need to change your tampon or sanitary towel?

The questionnaire results in a score that assesses heavy menstrual bleeding. Affirmative responses to questions one and three were assigned a weight of three points each, whereas affirmative responses to all remaining questions were assigned a weight of one point each. A total score of ≥3 is considered indicative of heavy menstrual bleeding [30]. To enhance the usability of the iron deficiency scoring system, the individual questions from the SAMANTA questionnaire were incorporated into its development.

The students were weighed and their height was measured, and non-fasting blood samples were collected for the analysis of B-haemoglobin and S-ferritin. For further methodological details, see the Iron Insight Study [28].

### Data analysis

To calculate p-values for comparing baseline characteristics, Student’s t-test was used for normally distributed continuous variables, Mann-Whitney U-test for non-normally distributed variables, and Chi-square test for categorical data.

Candidate variables were selected post hoc from the available questionnaire items based on clinical relevance and suitability for use in a screening context. These variables were entered directly into multivariable logistic regression models without a preceding univariable screening step. Backward multivariable logistic regression was then used to identify the variables most strongly associated with iron deficiency.

A risk score was subsequently calculated for all participants, with each included item contributing one point. Different weighting schemes based on odds ratios were explored to achieve higher sensitivity or specificity. A final logistic regression analysis was performed using iron deficiency as the dependent variable and the composite risk score as the independent variable. Hosmer-Lemeshow goodness of fit and Nagelkerke R^2^ were calculated to assess model performance.

Sensitivity, specificity, positive predictive value (PPV) and negative predictive value (NPV) were determined using cross-tabulation. Youden’s index (J = sensitivity + specificity − 1) was calculated at each step of the risk score to determine a suitable threshold value. A receiver operating characteristic (ROC) curve was plotted, and the area under the curve (AUC) was calculated to further evaluate the instrument’s predictive performance for iron deficiency. Statistical analyses were performed using IBM SPSS Statistics for Windows, Version 29·0 (IBM Corp., Armonk, NY, USA).

### Ethical considerations

The study was approved by the Swedish Ethical Review Authority (Dnr 2023-01088-01), and all participants provided written informed consent prior to inclusion. All participants received their blood test results. Those with pathological values, such as anaemia or high ferritin levels, were referred to a healthcare centre, while participants with iron deficiency without anaemia received written advice.

## Results

**Figure 1.**
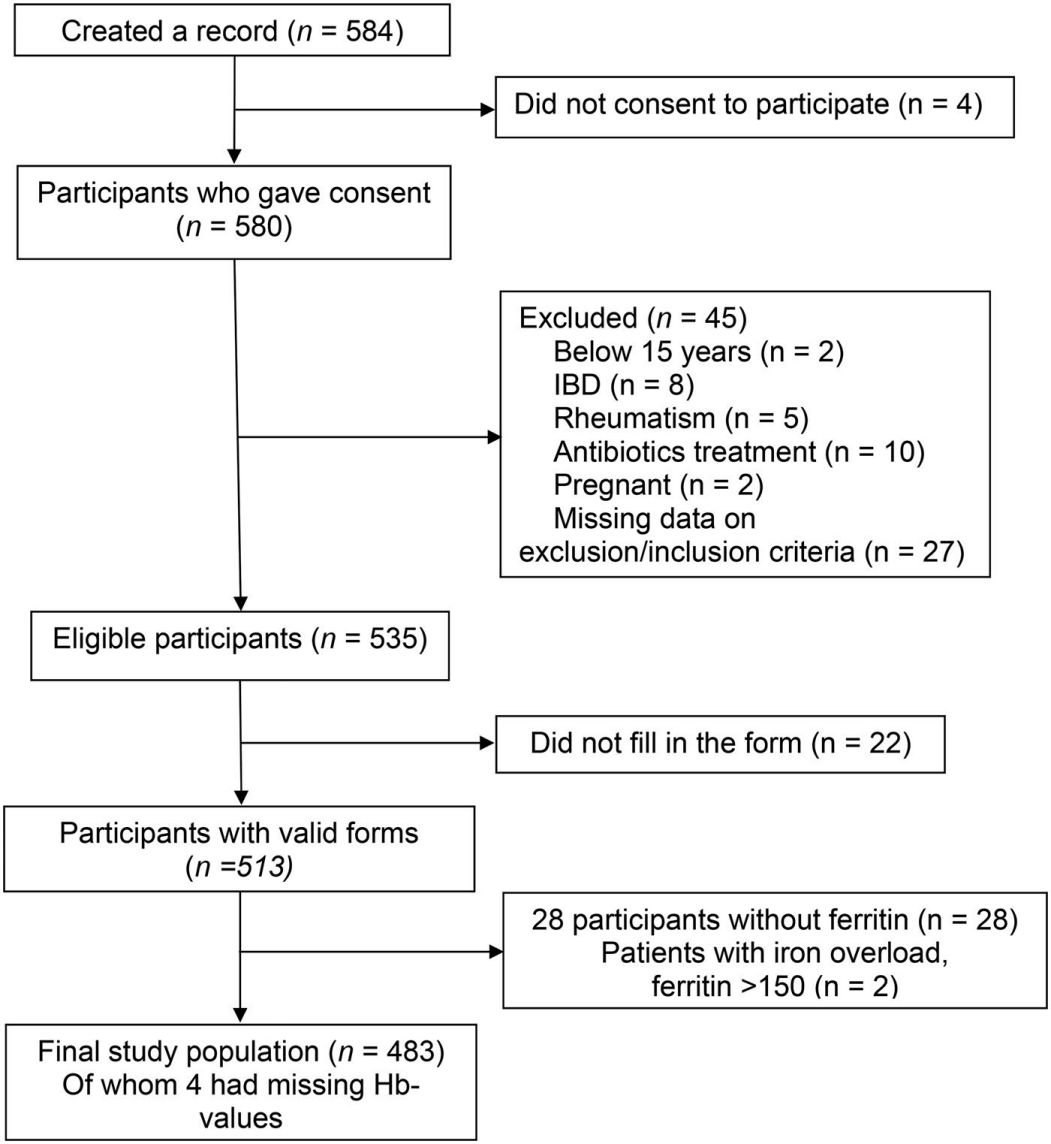
Flowchart illustrating the study inclusion process. A total of 584 adolescents were identified, of whom 580 provided informed consent. After exclusion of participants not meeting inclusion criteria, incomplete questionnaires, missing ferritin values, and iron overload (ferritin >150 µg/L), the final study population consisted of 483 participants.

A total of 580 individuals provided informed consent to participate in the study. 36 participants were excluded based on the exclusion criteria. Additionally, 31 did not complete the form, 28 had a missing ferritin value, and two had markedly elevated serum ferritin (>150 µg/L). As a result, the final study population consisted of 483 individuals of whom 183 were classified as iron deficient (serum ferritin <15 µg/L) and 300 as iron sufficient. Four participants had missing haemoglobin values, which did not affect classification of iron deficiency (Figure 1). Among participants with valid questionnaires, completion rates for the individual IRON-5 items exceeded 90%. Baseline characteristics, questionnaire responses, and group comparisons are presented in Table 1.

**Table 1.** Baseline characteristics.

	AllN = 483	Iron sufficient (ferritin ≥ 15) *n* = 300	Iron deficient (ferritin <15) *n* = 183	*P*-value
Age, years, mean (SD)	16·6 (0.9)	16·6 (0.9)	16·6 (0·9)	0·987
BMI, kg/m^2^, mean (SD)	21·7 (2·9)	21·8 (3·0)	21·5 (2·8)	0·668
Haemoglobin, g/L, mean (SD)	132·2 (10·5)	135·6 (7·8)	126·6 (11·9)	**<0·001**
Ferritin, µg/L, median (IQR)	19·0 (10–31)	27·0 (20–40)	8·0 (6–11)	**<0·001**
Anaemia*	37 (7·7)	5 (1·7)	32 (17·6)	**<0·001**
*SAMANTA heavy menstrual bleeding questionnaire*				
1. Bleeding more than 7 days	87 (18·1)	48 (16·1)	39 (21·4)	0·142
2. Bleeding heavily more than 3 days	299 (62·2)	164 (55·0)	135 (73·8)	**<0·001**
1. Heavy bleeding making me uncomfortable	138 (28·6)	67 (22·4)	71 (38·8)	**<0·001**
2. I get stains on clothes or sheets when bleeding heavily	191 (39·5)	103 (34·6)	88 (48·4)	**0·003**
3. I worry about getting stains on clothes or sheets when bleeding heavily	317 (65·9)	178 (59·7)	139 (76·0)	**<0·001**
4. I avoid activities, trips, or leisure when menstruating	186 (38·6)	94 (31·4)	92 (50·3)	**<0·001**
Non-omnivores	128 (26·5)	54 (18·0)	74 (40·4)	**<0·001**
No hormonal contraception	398 (82·6)	239 (79·7)	159 (87·4)	0·083
No iron supplement	430 (89·0)	261 (87·0)	169 (92·3)	0·163

Values are expressed as *n* (%) unless specified otherwise.*Hb <120 g/LThe *p*-value represents differences between the two groups (iron sufficient and iron deficient), calculated using Student’s t-test for normally distributed continuous variables, Mann-Whitney U-test for non-normally distributed variables, and Chi-square test for categorical data.

In the multivariable logistic regression model, the following variables were independently associated with iron deficiency (Table 2):

**Table 2.** Initial logistic regression model including candidate screening questions associated with iron deficiency.

Variable	OR	95% CI for OR	*P* value
Bleeding more than 7 days	0·981	0·571 to 1·687	0·945
I get stains on clothes or sheets when bleeding heavily	1·198	0·754 to 1·903	0·444
BMI <18.5	1·387	0·690 to 2·786	0·358
Bleeding heavily more than 3 days	1·517	0·948 to 2·428	0·082
I worry about getting stains on clothes or sheets when bleeding heavily	1·597	0·982 to 2·598	0·059
I avoid activities, trips, or leisure when menstruating	1·679	1·076 to 2·620	**0·022**
Heavy bleeding making me uncomfortable	1·698	1·025 to 2·813	**0·040**
No hormonal contraceptive	1·803	0·997 to 3·260	0·051
No iron supplement	2·655	1·288 to 5·473	**0·008**
Non-omnivores	3·887	2·444 to 6·182	**<0·001**

Hosmer-Lesmeshow goodness-of-fit test *p* value: 0·990. Nagelkerke R^2^ = 0·213. Significant *p* values are indicated in bold.

- Not being an omnivore (pescatarian, vegetarian/vegan, or non-consumers of red meat).
- Avoiding activities, trips, or leisure when menstruating.
- Heavy bleeding resulting in discomfort.
- No use of iron supplements or dietary supplements containing iron.

After conducting backward logistic regression, the following factors were identified as independently associated with iron deficiency and were included in the risk assessment instrument due to their suitability for use in a screening context (Table 3):

**Table 3.** Final logistic regression model of the included questions in IRON-5.

Variable	OR	95% CI for OR	*p* value
Not defined as omnivore	3·695	2·356 to 5·794	**<0·001**
No iron supplements	2·605	1·275 to 5·321	**0·009**
Heavy bleeding making me uncomfortable	2·301	1·465 to 3·615	**<0·001**
No hormonal contraceptives	2·126	1·205 to 3·751	**0·009**
I avoid activities, trips, or leisure when menstruating	1·975	1·302 to 2·997	**0·001**

Hosmer-Lesmeshow goodness-of-fit test *p* value: 0·532. Nagelkerke R^2^ = 0·186. Significant *p*-values are indicated in bold.

- Not being omnivore (pescatarian, vegetarian/vegan, or non-consumers of red meat).
- Avoiding activities, trips, or leisure when menstruating.
- Heavy bleeding, resulting in discomfort.
- No use of iron supplements or dietary supplements containing iron.
- No use of hormonal contraceptives.

These questions were selected because, in combination, they showed the strongest overall association with iron deficiency, while also being possible to formulate as simple yes/no items. This balance between predictive strength and practical feasibility was essential to ensure the instrument’s applicability in a screening context.

**Figure 2.**
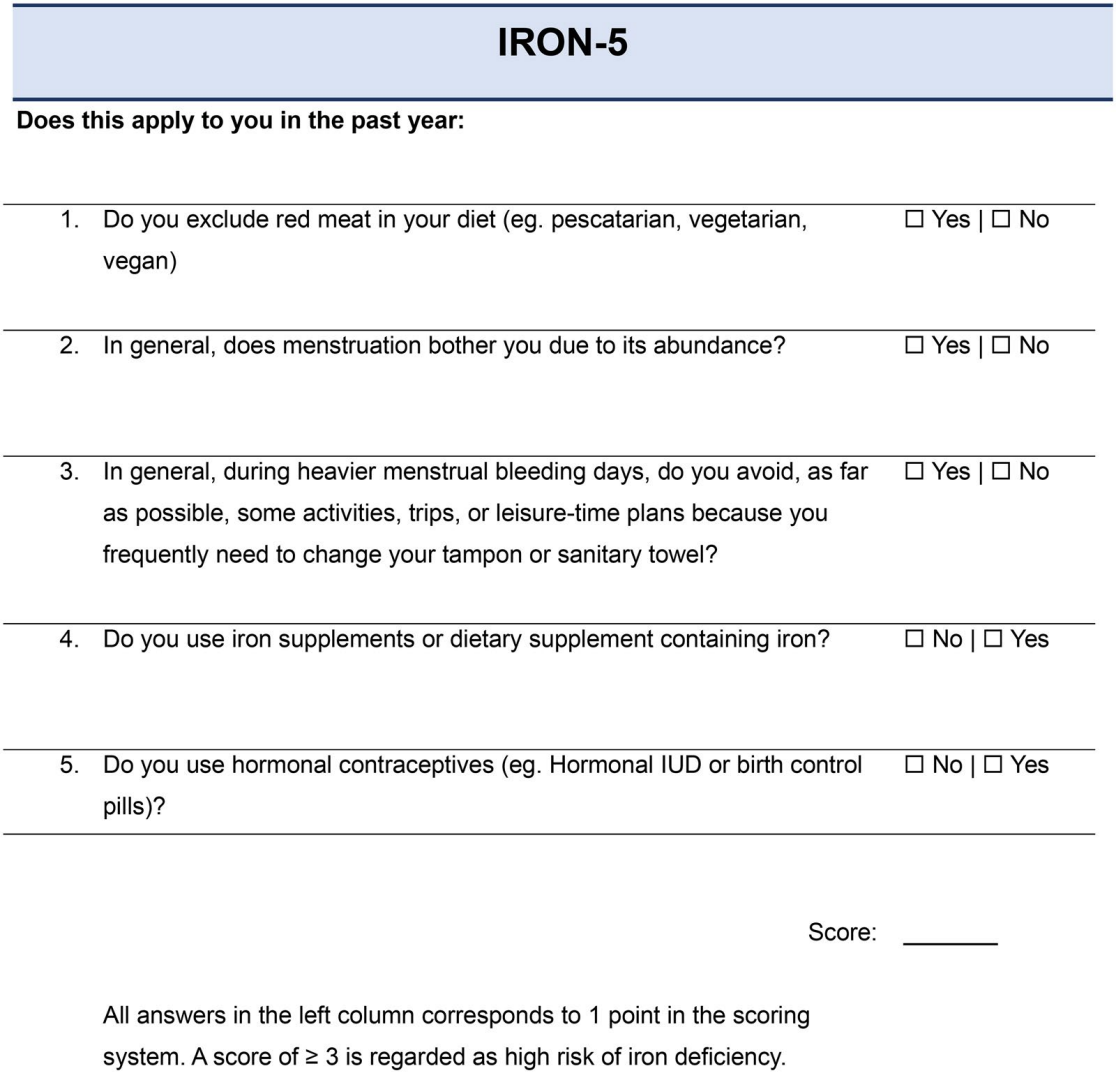
The IRON-5 screening questionnaire for identification of individuals at high risk of iron deficiency. The instrument consists of five yes/no items related to dietary habits, menstrual bleeding, activity limitation due to menstruation, iron supplementation, and hormonal contraceptive use. Each affirmative response scores one point; a total score ≥3 indicates high risk of iron deficiency.

A scoring system ranging from 0 to 5 points was developed based on the five selected questions, with one point assigned for each response indicating an increased risk of iron deficiency (Figure 2).

The area under the curve receiver operating characteristics (ROC) curve (AUC) was 0.723 (95% CI: 0.676–0.770). Youden’s index was calculated at each score level, indicating that a suitable score threshold would be three (Table 4). At this threshold, the risk assessment instrument demonstrated a sensitivity of 74%, specificity of 57%, positive predictive value of 51%, and a negative predictive value of 78%. This cut-off was considered optimal to maximize the screening instrument’s overall clinical utility.

**Table 4.** Sensitivity, specificity, and youden’s index were calculated for each cut-off in IRON-5.

Score	Sensitivity	Specificity	PPV*	NPV**	Youden’s Index
0	100	0·00	0·38	NA	0·00
1	1·00	0·01	0·38	1·0	0·01
2	0·96	0·12	0·40	0·82	0·08
**3**	**0·74**	**0·57**	**0·51**	**0·78**	**0·31**
4	0·33	0·91	0·70	0·69	0·24
5	0·08	1·00	0·93	0·64	0·08

*Positive Predictive Value. **Negative Predictive Value. NA – Not applicable.

**Figure 3.**
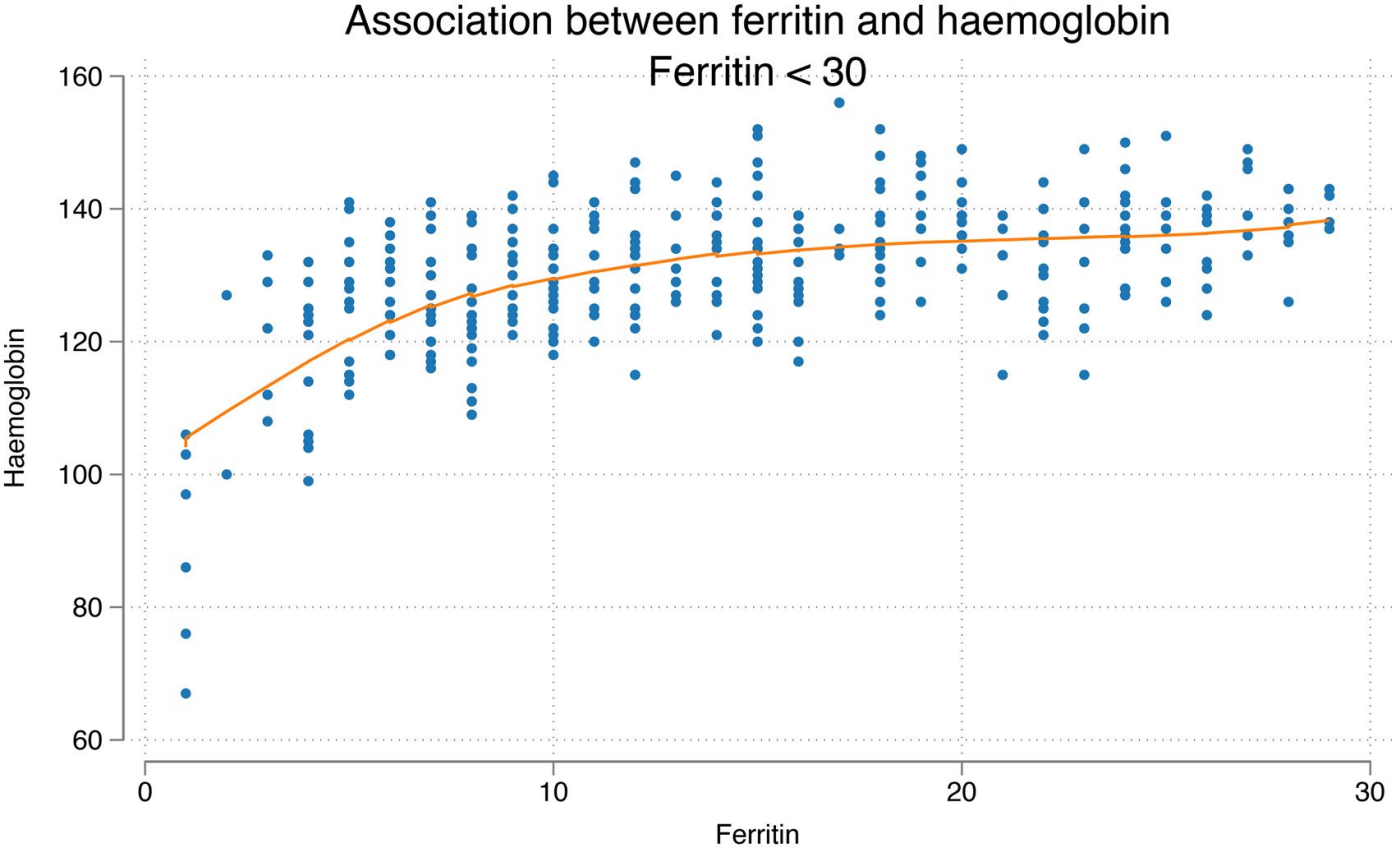
Scatterplot of ferritin versus haemoglobin with Lowess smoother (bandwidth = 0.8). A positive association between ferritin and haemoglobin is evident up to approximately 15 µg/L ferritin (*n* = 348).

**Figure 4.**
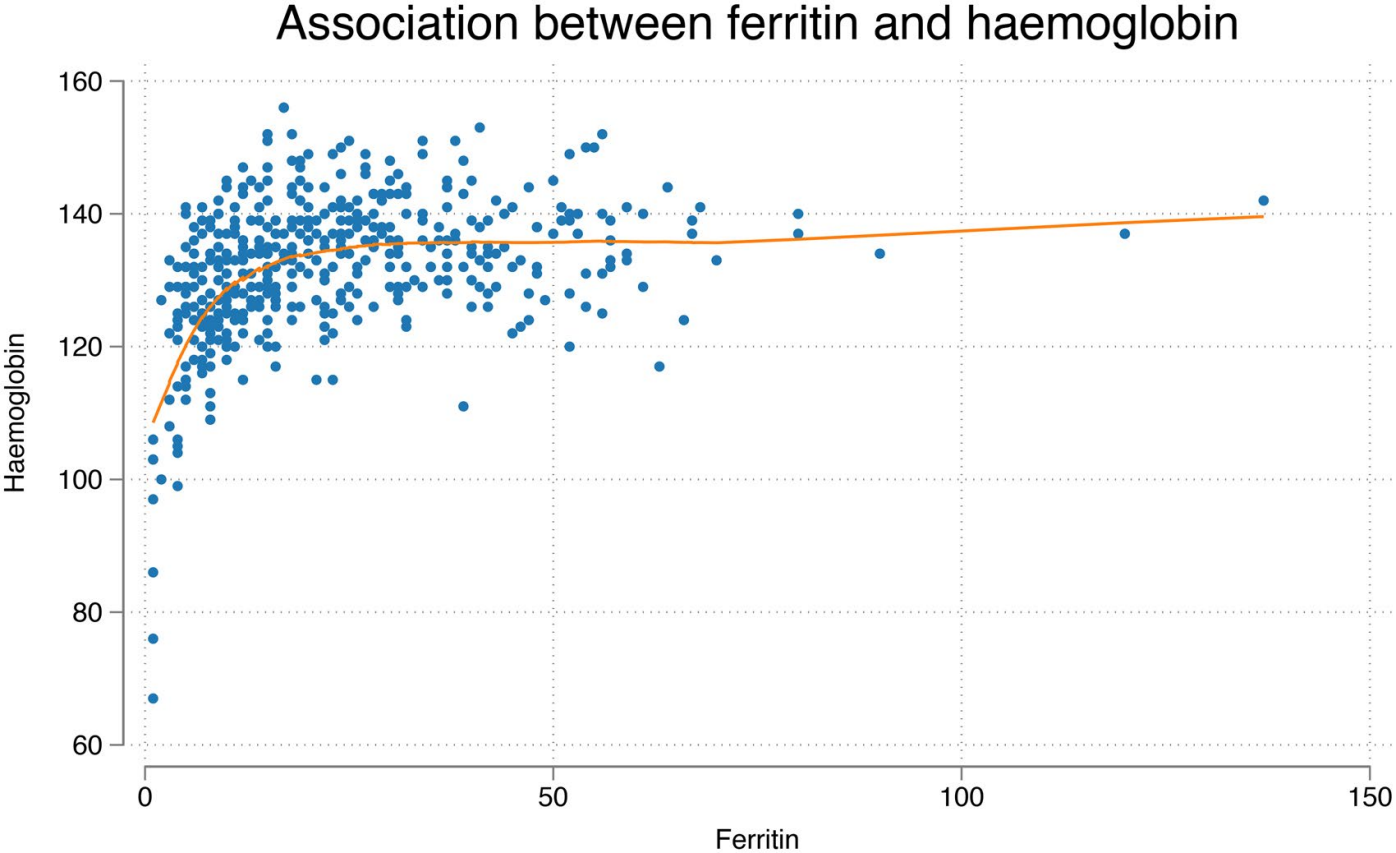
Scatterplot of ferritin versus haemoglobin with Lowess smoother (bandwidth = 0.8) for all participants.

Linear regression analysis showed a statistically significant positive association between ferritin and haemoglobin levels within the ferritin interval of 7–15 µg/L. Haemoglobin increased by 1.4 g/L for each µg/L increase in ferritin in this range (*p* < 0.001). In contrast, no significant correlation was observed between ferritin and haemoglobin at ferritin levels above 15 µg/L, suggesting that, in our sample, haemoglobin values plateau beyond this threshold (Figures 3 and 4).

## Discussion

Our five-item instrument, IRON-5, could be a useful tool for identifying young girls with iron deficiency. Importantly, haemoglobin was not included in the definition of the target condition, as iron deficiency may be present in the absence of anaemia and would otherwise be missed by haemoglobin-based screening. Ferritin reflects iron stores, and low ferritin has been shown to correspond to absent or markedly depleted bone-marrow iron in population-based studies, supporting its use as the primary marker of iron deficiency [31].

With a scoring system ranging from zero to five and a cut-off at three points, it achieves a sensitivity of 74% and a specificity of 57%. A higher score was also associated with an increased risk of iron deficiency, with a specificity of 91% at four points and 100% at five points. This could be compared to the widely used Centor criteria for diagnosing tonsillitis, which has a sensitivity of 49% and a specificity of 82% [32]. In our model, we chose to focus on a higher sensitivity with a moderate specificity to identify more individuals at risk. A risk-based questionnaire approach to prioritize confirmatory testing is broadly consistent with the Wilson and Jungner principles of screening; a detailed explanation is provided in Appendix A (supplementary material).

Some of the predictors included in the IRON-5 instrument are conceptually related, particularly those reflecting different dimensions of menstrual bleeding and its impact on daily activities. Such interrelationships may introduce correlation between predictors and affect the interpretability of individual regression coefficients. However, the aim of the present analysis was not causal inference but the development of a pragmatic screening tool. Correlated predictors were therefore retained when they contributed complementary, clinically meaningful information relevant to risk identification. In this context, overall model performance and screening characteristics were prioritized over isolated coefficient interpretation.

At the selected threshold of ≥3 points, IRON-5 demonstrates a sensitivity of 74% and a specificity of 57%, corresponding to a negative predictive value of 78% and a positive predictive value of 51% in this high-prevalence population. This implies that some individuals with iron deficiency will be missed, and that a proportion of those screening positive will not have iron deficiency. However, this level of misclassification must be interpreted in relation to the intended purpose of the instrument. IRON-5 is not designed to establish or exclude iron deficiency, but to prioritize individuals for confirmatory blood testing. In this context, false-positive results primarily lead to additional testing, while false-negative results highlight the importance of repeated assessment and clinical judgment rather than a single screening occasion [33].

Alternative cut-offs illustrate the inherent trade-off between sensitivity and specificity: lower thresholds achieve very high sensitivity but unacceptably low specificity, whereas higher thresholds markedly improve specificity at the expense of missing a large proportion of cases. The ≥3 cut-off was therefore considered a pragmatic balance between sensitivity and specificity for use as a screening instrument in settings with limited resources.

In the Swedish school system, every high school student meets with a nurse at least once during a period of three years, presenting an opportunity to administer this questionnaire and identify girls at high risk for iron deficiency. Early identification allows for timely interventions, such as iron supplements, dietary advice, or contraceptives, which can potentially enhance well-being, productivity, and academic performance in this group [11–14]. Additionally, establishing a confirmed diagnosis of iron deficiency prior to initiating treatment is crucial, as iron supplementation is frequently associated with gastrointestinal side effects, and may be harmful in individuals with undiagnosed hemochromatosis [34,35].

Even if low ferritin levels are common among young women and may, in some cases, represent a physiological state related to menstruation and dietary patterns, this does not preclude potential clinical relevance. At a population level, even modest improvements in iron status could plausibly translate into meaningful effects on well-being, fatigue, and physical or cognitive performance in a subgroup of individuals [11–14]. However, current evidence does not allow firm conclusions regarding which individuals are most likely to benefit from intervention, particularly among those identified through screening rather than clinical presentation. Further studies are therefore needed to determine the symptom burden associated with low ferritin in otherwise healthy young women and to identify which subgroups may derive clinically relevant benefit from treatment. Symptoms such as fatigue are common and often normalized among young women, where they may be attributed to ‘normal’ adolescent tiredness. In some individuals, however, such symptoms may reflect underlying iron deficiency and be potentially modifiable. This highlights the value of targeted assessment in selected individuals, while acknowledging that symptom-based attribution alone is insufficient.

Importantly, the evidence supporting symptomatic improvement with iron treatment in individuals with iron deficiency without anaemia largely originates from studies including patients who sought care due to symptoms, most commonly fatigue, and evidence for treatment benefit in truly asymptomatic, screening-detected populations remains limited. Consequently, the present study does not aim to establish treatment efficacy in asymptomatic individuals identified through screening, but rather to support identification of individuals at increased risk of iron deficiency who may benefit from confirmatory testing and individualized clinical assessment. The clinical and cost-effectiveness of treating screen-detected, asymptomatic iron deficiency warrants further investigation.

Prior studies have questioned the effectiveness of screening for iron deficiency based solely on risk factors, except in cases of heavy menstrual bleeding [36]. In the Iron Insight study, participants classified as omnivores had a higher ferritin level than those who excluded red meat. Since oral supplements are an effective and widely used treatment for iron deficiency, their use was considered an inverse risk factor [23,24,37].

Recent research has challenged the current WHO-defined serum ferritin threshold of <15 µg/L for diagnosing iron deficiency. In a large multinational meta-analysis, Addo and colleagues identified physiologically derived thresholds for the onset of iron-restricted erythropoiesis, reporting a consistent serum ferritin cut-off of <24.8 µg/L in non-pregnant women [38].

In our study, we defined iron deficiency according to the current WHO guideline of ferritin <15 µg/L. Although there is increasing support for adopting physiologically based thresholds around 25 µg/L, we observed no appreciable decline in haemoglobin concentrations among participants with ferritin levels above 15 µg/L. We therefore consider the WHO definition to be a pragmatic and appropriate cut-off for our study population. Nevertheless, we acknowledge that the use of higher, physiology-informed thresholds may enhance the sensitivity of screening efforts and enable earlier detection of iron deficiency.

## Strengths and limitations

The screening instrument is easy to use, can be applied quickly, and could be integrated into existing routine examinations of young women in school health programs. The questions included in the screening instrument showed high completion rates among participants (>90%), and, apart from weight, the study had little missing data.

Ferritin, while an indirect marker of iron stores, is also an acute-phase reactant and may be elevated due to ongoing inflammation, infections, autoimmune disorders, and obesity [39]. To account for this we excluded girls with chronic inflammatory diseases. Ongoing infection was assessed by self-reported current antibiotic treatment; however, no inflammatory markers such as CRP were measured, and participants were not asked about recent febrile illness or upper respiratory infection. Consequently, some participants with recent infections or mild infections not requiring antibiotic treatment may have had falsely elevated serum ferritin levels and were not identified by the exclusion criteria.

Girls with obesity were included due to the high prevalence of obesity and its relatively modest contribution to systemic inflammation. Ferritin levels may also be elevated in patients with anorexia nervosa, potentially leading to an underestimation of BMI as a risk factor [40]. Although a weak association between low BMI and iron deficiency was observed, it was not statistically significant, and adjusting for weight did not alter the results. Including additional biomarkers such as CRP or transferrin saturation (TSAT) could have improved diagnostic accuracy; however, the current approach reflects a common clinical setting where such markers are not routinely assessed.

In addition, a small number of participants with markedly elevated serum ferritin levels were excluded to reduce the risk of misclassification. This was a pragmatic decision, as markedly elevated ferritin may reflect inflammatory or infectious processes rather than true iron excess. Nevertheless, elevated ferritin due to recent infection cannot be entirely ruled out, and exclusion based on ferritin levels may have contributed to a small degree of outcome misclassification.

Another limitation is that this screening instrument was developed for a specific population using self-reported data, and its applicability to other age groups remains unknown. It is also not usable for those who do not menstruate, such as men or pre-/postmenopausal women. While this instrument may offer a practical tool for identifying individuals at high risk, further research is necessary to validate its predictive validity.

It is important to acknowledge potential biases in our study. Participants were informed that the study focused on fatigue and iron deficiency, which may have attracted individuals already experiencing tiredness, and potentially affected the results. At this stage, we cannot determine the questionnaire’s overall usefulness or the potential benefits of identifying girls with iron deficiency. Future research should assess both the costs and benefits of implementing such a screening instrument.

The results of this study provide valuable insights into the risk factors associated with iron deficiency among adolescent girls. Iron deficiency is significantly more prevalent among girls than boys and may contribute to lower perceived health in this group [5,6]. While a meta-analysis confirmed a significant therapeutic effect of iron supplementation in patients with fatigue and iron deficiency, it did not establish a clear association between iron deficiency and fatigue [8]. These contradictory results indicate a lack of knowledge and should be further investigated. Another important topic is adherence to treatment and recommendations, as well as the effectiveness of treatment recommendations, which could also be a focus of future research. Addressing iron deficiency remains crucial, especially considering that reduced meat consumption, while beneficial for both environmental sustainability and public health, may also contribute to an increased prevalence of iron deficiency [9,41].

## Conclusions

Our findings suggest that IRON-5, a questionnaire consisting of five items, could facilitate early identification of iron deficiency in adolescent girls. As universal blood testing to screen for iron deficiency in this population is unlikely to be feasible in most healthcare systems, a short, questionnaire-based tool may offer a practical, resource-efficient alternative. This study addresses an important evidence gap by introducing a structured, non-invasive screening instrument to guide targeted diagnostic testing in clinical and preventive settings.

Current screening practices often rely on haemoglobin testing, which detects anaemia but does not identify iron deficiency in the absence of anaemia. Consequently, a substantial proportion of iron-deficient individuals may remain undetected. The present results indicate that specific self-reported characteristics can help identify those at higher risk. While IRON-5 may support earlier recognition and more targeted management of iron deficiency in primary care and school health services, further validation of the instrument is required. In addition, further research is needed to evaluate the potential for overdiagnosis and to determine whether treatment of asymptomatic, screening-detected iron deficiency provides clinically meaningful benefits before broader implementation of such a screening approach can be recommended.

## Supplementary Material

Appendix_A.docx
