# Supplementary material for: Screening for iron deficiency in young women: the predictive validity of a five-item screening instrument (IRON-5)
Source: Scand J Prim Health Care. 2026 Mar 31;44(1):2649329. doi: 10.1080/02813432.2026.2649329 (PMC13047845; doi:10.1080/02813432.2026.2649329)
Supplement: Appendix_A.docx [file IPRI_A_2649329_SM4673.docx]

**Appendix A**

**Wilson and Jungner screening principles and the proposed IRON-5 approach**

**Important health problem.** Iron deficiency is common in adolescent girls and is associated with impaired well-being, physical performance, and cognitive function even in the absence of anaemia.
**Recognizable latent stage.** Iron deficiency typically develops gradually and may be present without anaemia or overt clinical signs; serum ferritin allows detection before iron-deficiency anaemia develops.
**Natural history understood.** The progression from depleted iron stores to iron-restricted erythropoiesis and anaemia is well described, with menstrual blood loss and low intake of bioavailable iron as key contributors in this age group.
**Accepted treatment.** Iron deficiency can be treated effectively with oral iron supplementation and/or dietary interventions; management of heavy menstrual bleeding (e.g., hormonal contraception) can reduce ongoing iron loss.
**Facilities for diagnosis and treatment.** Confirmatory blood testing (ferritin) and first-line interventions (dietary counselling, oral iron, contraceptive counselling when appropriate) are available in routine primary care and school health services.
**Suitable test.** IRON-5 is a brief, five-item questionnaire intended to identify individuals at higher risk and prioritize them for confirmatory testing; it is simple and quick to administer and showed acceptable discrimination in this cohort.
**Acceptable test.** The instrument is non-invasive and consists of five yes/no questions, making it likely acceptable in school and primary care settings.
**Agreed policy on whom to treat.** IRON-5 is not intended to guide treatment directly; individuals above the threshold should be offered confirmatory blood testing prior to any intervention. Clear follow-up pathways and treatment thresholds should be aligned with existing clinical practice and evolving ferritin cut-offs; this is an implementation aspect requiring further study.
**Economic balance.** The questionnaire approach has minimal direct cost and may reduce unnecessary blood testing; however, formal cost-effectiveness analyses are needed before large-scale implementation.
**Continuous process.** In Sweden, adolescents are routinely seen in school health services, which enables repeated administration over time (e.g., annually or when symptoms/risk factors arise), supporting a programmatic rather than one-time approach.
